# CX3CR1^+^ age-associated CD4^+^ T cells contribute to synovial inflammation in late-onset rheumatoid arthritis

**DOI:** 10.1186/s41232-025-00367-4

**Published:** 2025-02-06

**Authors:** Mitsuhiro Akiyama, Sohma Wakasugi, Keiko Yoshimoto, Koichi Saito, Sho Ishigaki, Risa Inukai, Yoshiyuki Matsuno, Waleed Alshehri, Yasushi Kondo, Yuko Kaneko

**Affiliations:** Division of Rheumatology, Department of Internal Medicine, Keio University School of Medicine, 35 Shinanomachi, Shinjuku-Ku, Tokyo, 160-8582 Japan

**Keywords:** CX3CR1, Cytotoxic T cells, Aging, Rheumatoid arthritis, Late-onset rheumatoid arthritis, Difficult-to-treat rheumatoid arthritis

## Abstract

**Background:**

Recent evidence suggests that clonally expanded cytotoxic T cells play a role in various autoimmune diseases. Late-onset rheumatoid arthritis (LORA) exhibits unique characteristics compared to other RA forms, suggesting distinct immunological mechanisms. This study aimed to examine the involvement of cytotoxic T cells in LORA.

**Methods:**

Fresh peripheral blood samples were collected from 78 treatment-naïve active RA patients, 12 with difficult-to-treat RA, and 16 healthy controls. Flow cytometry was employed to measure the proportions of CX3CR1^+^cytotoxic CD4^+^ and CD8^+^ T cells in these samples. Additionally, immunohistochemical staining was performed on lymphoid node and synovial biopsy samples from patients with RA.

**Results:**

CX3CR1^+^cytotoxic CD4^+^ T cells were specifically increased in untreated, active patients with LORA, displaying features of CXCR3^mid^ age-associated T helper cells known as “ThA”. CX3CR1⁺CD4⁺ T cells were identified as a cytotoxic ThA subset, as nearly all of these cells specifically expressed granzyme B. These cells were observed in enlarged lymph nodes and were found to infiltrate synovial tissues from patients with LORA. The proportions of CX3CR1^+^CD4^+^ T cells positively correlated with arthritis activity in LORA. The number of cells decreased after treatment with methotrexate, tumor necrosis factor inhibitors, and interleukin-6 inhibitors, whereas T-cell activation modulators did not affect them. Moreover, PD-1^+^CD38^+^CX3CR1^+^CD4^+^ T cells were identified as a treatment-resistant T cell subset that was characteristically increased in difficult-to-treat RA. CX3CR1^+^CD8^+^ T cells showed no significant difference between RA patients and healthy individuals, and no correlation with disease activity was observed. However, a correlation with age was observed in RA patients.

**Conclusions:**

Our findings suggest that the immunopathogenesis of RA differs by age of onset, with CX3CR1^+^ age-associated cytotoxic CD4^+^ T cells playing a significant role in LORA. Additionally, the presence of a specific CX3CR1^+^ T cell subset may be linked to treatment resistance.

**Supplementary Information:**

The online version contains supplementary material available at 10.1186/s41232-025-00367-4.

## Background

Rheumatoid arthritis (RA), which affects approximately 1% of the population, is an autoimmune disease characterized by the erosion of articular cartilage and bone, culminating in physical disability, persistent pain, and diminished quality of life [1–4]. Despite the transformative impact of biologics in RA treatment, a significant proportion of patients, ranging from 30 to 40%, fail to respond adequately, underscoring the urgent need for innovative therapeutic targets [5]. The involvement of CD4^+^ T cells in the pathogenesis of RA is highlighted by their genetic association with HLA-DRB1 [6, 7]. Various CD4^+^ T cell subsets, including T peripheral helper (Tph) cells with B-cell helper ability, have been identified within the synovial tissues and blood of patients with RA [8, 9]. Furthermore, recent studies using single-cell RNA sequencing have revealed the clonal expansion of CD4^+^ T cells exhibiting cytotoxic features in the blood and synovial fluid of patients with RA [10, 11]. This suggests that cytotoxic CD4^+^ T cells may contribute to the pathogenesis of RA.

In recent years, with the aging population, an increasing frequency of autoimmune diseases has emerged in elderly individuals. There are noticeable clinical differences between those who develop RA later in life and those who manifest it at younger ages. Patients with late-onset RA (LORA) tend to progress more acutely, with more frequent seronegativity and higher levels of serum inflammatory markers than patients with non-LORA [12]. Therefore, distinct immunological mechanisms may exist between LORA and non-LORA, although these differences have not been fully elucidated.

Here, we demonstrate that patients with untreated, active LORA exhibit an increase in CX3CR1^+^ cytotoxic CD4^+^ T cells, showing characteristics of CXCR3^mid^ age-associated T helper cells known as ThA. These cells were observed in enlarged lymph nodes and were found to infiltrate synovial tissues from patients with LORA. They correlated with disease activity and diminished with treatment with methotrexate, tumor necrosis factor inhibitors (TNFi), and interleukin-6 inhibitors (IL-6i), yet remained unaffected by T-cell activation modulators. Of note, despite multidrug therapy, PD-1^+^CD38^+^CX3CR1^+^ cytotoxic CD4^+^ T cell subset was persistently elevated in patients with difficult-to-treat RA (D2T RA), a recently recognized treatment-resistant phenotype. Thus, our findings suggest that CX3CR1^+^ cytotoxic CD4^+^ T cells could be potential targets for the management of LORA.

## Materials and methods

### Patients and controls

The study included 78 treatment-naïve patients with RA with active disease, 12 patients with D2T RA, and 16 healthy controls. Supplementary Table 1 illustrates the characteristics of the patients and healthy controls. All patients with RA met the 1987 American College of Rheumatology [13] or 2010 American College of Rheumatology/European League Against Rheumatism (EULAR) classification criteria [14]. Patients with an age of onset of 60 years or older were classified as LORA according to the previous studies [15]. D2T RA was defined according to EULAR criteria as patients who failed to respond to treatment with two or more biologic/targeted synthetic disease-modifying antirheumatic drugs with different modes of action [16]. Characteristics of D2T RA patients are shown in Supplementary Table 2. Non-D2T RA was defined as not meeting the criteria for D2T RA and achieving low disease activity or remission by clinical disease activity index (CDAI) 24 weeks after treatment initiation. Approval for the study was granted by the Ethics Committee of Keio University School of Medicine (Approval number: 20140335). The study was conducted in accordance with the principles outlined in the Declaration of Helsinki. Written informed consent was obtained from all the participants.

### Flow cytometric analysis

Flow cytometric analysis was conducted using whole blood samples freshly obtained from patients and healthy controls, following the protocols recommended by the manufacturers. The antibodies used in this study are listed in Supplementary Table 3. A 30-min incubation of 50 μL of whole blood sample was conducted at room temperature with fluorescence-labeled antibodies. Subsequently, the cells were lysed and fixed using Lyse/Fix Buffer (BD Biosciences, San Jose, CA, USA). For intracellular staining of granzyme B (GZMB), or phosphorylated signal transducer and activator of transcription 1 (STAT1), cells were permeabilized and fixed using reagents from the BD Pharmingen™ Transcription Factor Buffer Set, followed by a 30-min incubation with antibodies. Cells were then washed and analyzed using the MACSQuant Analyzer (Miltenyi Biotec, Bergisch Gladbach, Germany) or BD LSRFortessa™ (BD Biosciences, San Jose, CA, USA). Data were analyzed using the FlowJo software v10 (Tree Star, Stanford University, CA, USA). Single-cell mapping was conducted using a t-distributed Stochastic Neighbor Embedding (tSNE) algorithm [17].

### Immunohistochemistry

Immunohistochemistry was performed on paraffin-embedded synovial and lymph node tissue sections obtained from patients with RA. Deparaffinization, antigen retrieval (20 min in citrate buffer, pH 6.0), primary antibody incubation (30 min), peroxide blocking (5 min), DAB staining (two rounds of 5 min each), and hematoxylin staining (5 min) were performed using an automated BOND immunostaining system. The primary antibody used was a CX3CR1 polyclonal antibody (Proteintech Group, Inc., #13,885–1-AP), applied at a dilution of 1:50.

### Statistical analysis

Data were analyzed using the GraphPad Prism software (v7; GraphPad Software, La Jolla, CA, USA). For comparisons between two groups of unpaired samples, the Mann–Whitney *U* test or *t*-test was used based on whether the distribution of data was non-parametric or parametric. For paired samples, the Wilcoxon matched-pairs signed rank test or paired *t*-test was used based on the non-parametric or parametric distribution of data. To compare more than two groups, a one-way ANOVA followed by Tukey's multiple comparison test was conducted. Fisher's exact test was used for categorical analyses. Spearman's rank correlation coefficient was used for the correlation analysis. Statistical significance was defined as a two-sided *p*-value of less than 0.05.

## Results

### *Peripheral blood CX3CR1*^+^*cytotoxic T cells are increased in LORA*

**Fig. 1:**
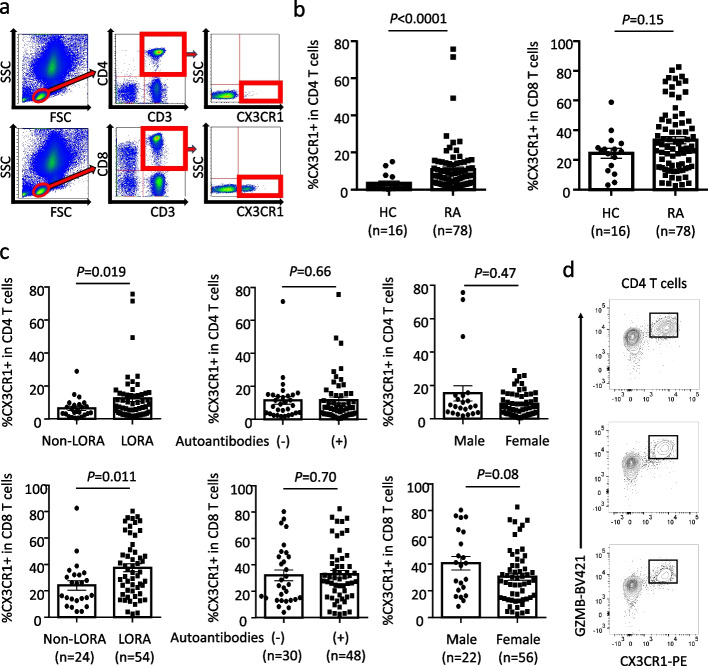
Peripheral blood CX3CR1^+^ T cells are increased in LORA. **a** Gating strategy for identifying CX3CR1^+^ T cells by flow cytometry. **b** Comparison of peripheral blood CX3CR1^+^ T cells between treatment-naive, active RA, and HC. Mann–Whitney U test. **c** Comparative analysis of peripheral blood CX3CR1^+^ T cells among clinical characteristics of RA (LORA, seropositivity, and sex difference). Seropositivity for autoantibodies refers to the presence of either RF, anti-CCP antibodies, or both. Mann–Whitney U test. **d** Intracellular GZMB expression in CX3CR1⁺CD4⁺ T cells. The area within the black bold frame represents CX3CR1⁺GZMB⁺CD4⁺ T cells

In a previous study, we reported that CX3CR1^+^ T cells specifically express GZMB and perforin, indicating a cytotoxic T cell subset [18]. We found that the proportion of CX3CR1^+^CD4^+^ T cells was elevated in the peripheral blood of 78 patients with active, treatment-naïve RA compared with that in healthy controls (Fig. 1a, b). However, there was no difference in the proportion of CX3CR1^+^CD8^+^ T cells between patients with RA and healthy controls (Fig. 1b). Subsequently, we investigated the relationship between the clinical features of RA and the elevated proportion of CX3CR1^+^ T cells. We found that in patients with active, treatment-naïve LORA, the proportions of both CX3CR1^+^CD4^+^ T cells and CX3CR1^+^CD8^+^ T cells were significantly increased compared to those in patients with non-LORA (Fig. 1c). As shown in supplementary Table 4, LORA patients had significantly higher levels of inflammatory markers, including erythrocyte sedimentation rate and C-reactive protein, and exhibited higher disease activity compared to non-LORA patients. Autoantibodies, including rheumatoid factor and/or anti-cyclic citrullinated peptide antibodies, which are observed in approximately 70% of patients with RA [19], were also observed in the RA cohort included in our study (Supplementary Table 1). We performed a comparative analysis based on the presence of autoantibodies or sex of the patients but found no association between these factors and the proportion of CX3CR1^+^ T cells (Fig. 1c). We further performed a comparative analysis of CX3CR1⁺ T cells based on the presence or absence of autoantibodies in both the LORA and non-LORA groups. As a result, no differences were observed in the proportion of CX3CR1⁺ T cells between autoantibody-positive and autoantibody-negative patients in either group (Supplementary Fig. 1). Next, to investigate whether CX3CR1⁺CD4⁺ T cells in LORA patients possess cytotoxic function, we examined the expression of GZMB in these cells. We found that nearly all CX3CR1⁺CD4⁺ T cells in LORA patients expressed GZMB (Fig. 1d). Overall, these findings suggest a characteristic increase in the proportion of peripheral blood CX3CR1^+^ cytotoxic CD4^+^ T cells in patients with LORA.

### *CX3CR1*^+^*T cells in LORA exhibit molecular characteristics with age-associated T cells*

**Fig. 2:**
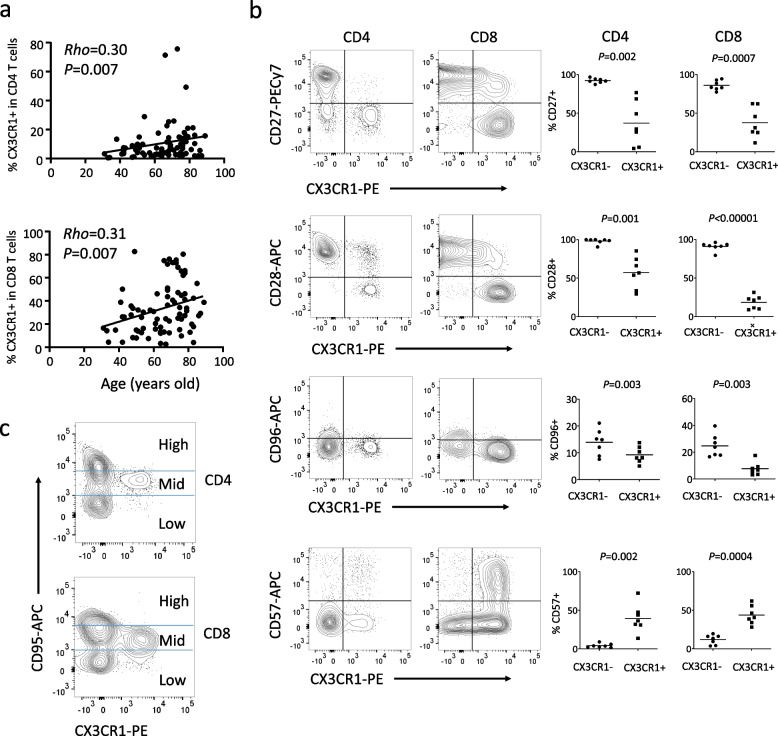
CX3CR1^+^ T cells exhibit characteristics of age-associated T cells. **a** Correlation analysis between the proportion of CX3CR1^+^ T cells and age in patients with RA (n = 78). Spearman’s correlation test. **b** Flow cytometry analysis of the expression of age-associated molecules (CD27, CD28, CD96, CD57) in CX3CR1^+^ T cells from peripheral blood of LORA patients (n = 7). Paired t-test. **c** Analysis of CD95 expression in CX3CR1^+^ T cells from peripheral blood of LORA

Given the discovery of an increased proportion of CX3CR1^+^ T cells in patients with LORA, we conducted an analysis to determine any correlation between age and the proportion of CX3CR1^+^ T cells in patients with active, treatment-naïve RA (n = 78). The proportions of CX3CR1⁺CD4⁺ T cells and CX3CR1⁺CD8⁺ T cells exhibited a mild but significant positive correlation with age (Fig. 2a, ρ = 0.3 and 0.31, respectively). On the other hand, there was no correlation between the proportion of CX3CR1^+^ T cells and age in healthy controls. Reportedly, age-associated T cells exhibit molecular characteristics such as decreased expression of CD27, CD28, and CD96, and increased expression of CD57 [20–22]. We examined whether CX3CR1^+^ T cells exhibited the molecular characteristics of age-associated T cells using seven blood samples from patients with active, treatment-naïve LORA. CX3CR1^+^CD4^+^ T cells and CX3CR1^+^CD8^+^ T cells were found to have low expression of CD27, CD28, and CD96 and high expression of CD57, indicating the molecular characteristics of age-associated T cells (Fig. 2b). In addition, we found that CX3CR1^+^CD4^+^ T cells and CX3CR1^+^CD8^+^ T cells exhibit a "mid" level of expression of CD95, which is associated with apoptosis [23] (Fig. 2c).

### *CX3CR1*^+^*T cells in LORA exhibit characteristics of CXCR3*^*mid*^* age-associated chemokine receptor expression*

**Fig. 3:**
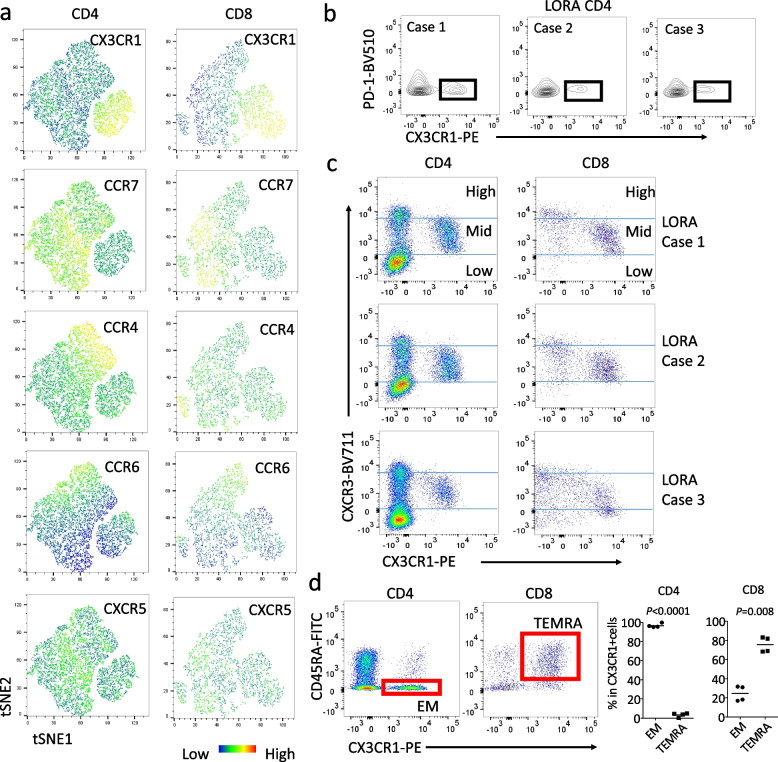
CX3CR1^+^ T cells exhibit features of CXCR3^mid^ age-associated chemokine receptor expression. **a** Unbiased analysis using flow cytometry and tSNE of chemokine receptor expression (CCR7 for lymph node-related, CCR4 for Th2 or Treg-related, CCR6 for Th17-related, CXCR5 for Tfh-related) in CX3CR1^+^ T cells. **b** Analysis of PD-1 expression in CX3CR1⁺CD4⁺ T cells from patients with LORA (n = 3). **c** Analysis of chemokine receptor expression related to Th1 (CXCR3^high^) or age-associated (CXCR3^mid^). **d** Phenotypic analysis of CX3CR1^+^ CD4^+^ or CX3CR1^+^ CD8^+^ T cells (EM or TEMRA). Paired t-test

To investigate whether CX3CR1^+^ T cells in LORA differed from classical Th1, Th2, Th17, Treg, and Tfh cells [24], we conducted a thorough analysis of chemokine receptor expression using an unbiased single-cell mapping approach with blood samples from four patients diagnosed with active, treatment-naïve LORA. CX3CR1^+^ T cells did not express CCR4 (Th2 or Treg marker), CCR6 (Th17 marker), or CXCR5 (Tfh marker), indicating an independent T cell subset (Fig. 3a). We also confirmed in the two-dimensional plots that CX3CR1^+^ T cells do not express the respective chemokine receptors (Supplementary Fig. 2). Furthermore, we investigated the expression of CD25 as an alternative Treg marker, but CX3CR1⁺CD4⁺ T cells derived from LORA patients did not express CD25 (Supplementary Fig. 3). We examined the expression of PD-1 in CX3CR1⁺CD4⁺ T cells derived from LORA patients, and our results showed that these cells did not express PD-1 (Fig. 3b). This finding indicates that CX3CR1⁺CD4⁺ T cells are distinct from Tph cells, which are known to exhibit high PD-1 expression [8]. Thus, the cells observed in our study are not part of the Tph cell population.

A recent report indicated that a population of cells with “mid” expression of CXCR3 (Th1 marker) represents age-associated helper T cells, known as “ThA” [25]. We discovered that all CX3CR1^+^CD4^+^ T cells were CXCR3^mid^ (Fig. 3c). On the other hand, only a subset of CXCR3^mid^ T cells (ThA) were CX3CR1^+^ (Fig. 3c). Considering that CX3CR1^+^CD4^+^ T cells specifically express GZMB (Fig. 1d), CX3CR1^+^CD4^+^ T cells represent a cytotoxic ThA subset. Additionally, we newly identified that CX3CR1^+^CD8^+^ T cells also exhibit CXCR3^mid^ expression (Fig. 3c). Examination of the phenotypic differences between CX3CR1^+^CD4^+^ T cells and CX3CR1^+^CD8^+^ T cells revealed that CX3CR1^+^CD4^+^ T cells are CCR7^−^CD45RA^−^effector memory (EM) cells, whereas CX3CR1^+^CD8^+^ T cells are terminally differentiated effector memory T cells re-expressing CD45RA (TEMRA) (Fig. 3d). Importantly, neither CX3CR1^+^CD4^+^ T cells nor CX3CR1^+^CD8^+^ T cells expressed CCR7, suggesting the possibility that both cell populations might have migrated from the lymph nodes to the peripheral blood (Fig. 3a, Supplementary Fig. 2).

### *CX3CR1*^+^*T cells in LORA differentiate in lymph nodes and contribute to inflammation in the inflamed synovial lesions*

**Fig. 4:**
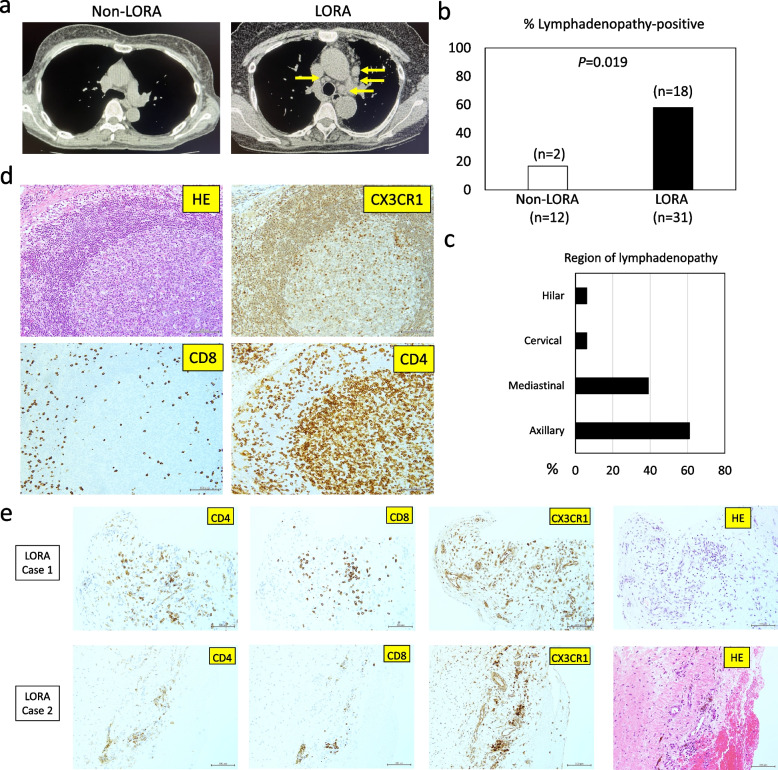
Identification of CX3CR1^+^ T cells in enlarged lymph nodes and synovial inflammatory sites. **a**,** b** Comparative analysis of the proportion of patients with lymphadenopathy on CT imaging in LORA and non-LORA groups. Fisher’s exact test. **c** Distribution of enlarged lymph nodes. **d** Immunostaining of CX3CR1^+^ CD4^+^ T cells and CX3CR1^+^ CD8^+^ T cells in lymph nodes. e. Immunostaining of CX3CR1^+^ CD4^+^ T cells and CX3CR1^+^ CD8^+^ T cells in synovial inflammatory tissues from LORA patients. Scale bar shows 100 μm

To explore the possibility that CX3CR1^+^ T cells in patients with LORA might undergo differentiation in the lymph nodes and subsequently migrate into the peripheral blood and synovial tissues, we investigated whether lymphadenopathy could be observed in patients with LORA. The proportion of patients with lymphadenopathy was significantly higher in the LORA group than in the non-LORA group (Fig. 4a, b). The majority of the lymphadenopathy regions were located in the axillary or mediastinal lymph nodes (Fig. 4c). Furthermore, immunohistochemical staining of enlarged lymph nodes showed the presence of CX3CR1^+^CD4^+^ T cells and CX3CR1^+^CD8^+^ T cells both inside and outside the lymphoid follicles (Fig. 4d). In addition, infiltration of CX3CR1^+^CD4^+^ T cells and CX3CR1^+^CD8^+^ T cells was detected at the synovial inflammation site in patients with LORA (Fig. 4e).

### *CX3CR1*^+^*CD4*^+^*T cells correlate with disease activity in LORA*

**Fig. 5:**
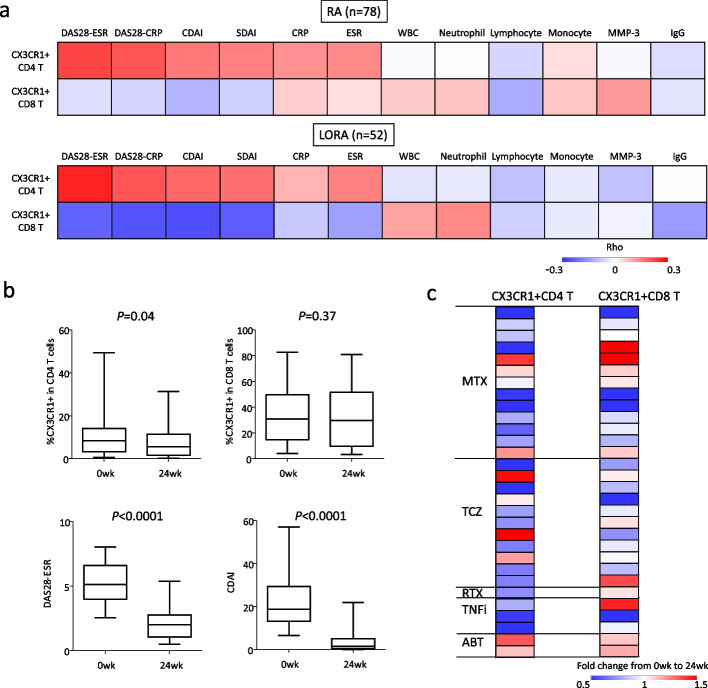
Association of disease activity with CX3CR1^+^ T cells and impact of treatment. **a** Heatmap of correlation analysis between peripheral blood CX3CR1^+^ CD4^+^ T cells, CX3CR1^+^ CD8^+^ T cells, and clinical indicators. Spearman's correlation analysis. **b** Longitudinal changes of CX3CR1^+^ T cells after initiation of treatment (n = 30). Wilcoxon's rank sum test. **c** Heatmap showing longitudinal changes of CX3CR1^+^ CD4^+^ T cells and CX3CR1^+^ CD8^+^ T cells analyzed based on specific treatments

Disease activity score 28-joint count (DAS28), CDAI, and simplified disease activity index (SDAI) are disease activity indices used to assess arthritis severity [26, 27]. These indices are critical for evaluating disease activity and treatment responses in patients with RA. We analyzed the correlation between CX3CR1^+^ T cells and disease activity indices in patients with active, treatment-naïve RA (*n* = 78). The proportion of CX3CR1^+^CD4^+^ T cells was positively correlated with arthritis activity and inflammatory marker levels (Fig. 5a). This was consistent even in the subgroup analysis that focused on 52 patients with active, treatment-naïve LORA (Fig. 5a). We note a stronger association of CX3CR1^+^CD4^+^ T cells with age than with disease activity, with correlation coefficients of 0.30, 0.22, and 0.16 for age, DAS28-ESR, and CDAI, respectively. In contrast, the proportion of CX3CR1^+^CD8^+^ T cells did not correlate with disease activity (Fig. 5a). Subsequently, the dynamics of CX3CR1^+^ T cells following the initiation of treatment with methotrexate or biologics were tracked over time in 30 patients with LORA. Along with an improvement in arthritis activity, the proportion of CX3CR1^+^CD4^+^ T cells decreased 24 weeks after treatment initiation (Fig. 5b). In contrast, there was no significant change in the proportion of CX3CR1^+^CD8^+^ T cells (Fig. 5b). In the analysis based on treatment types, methotrexate, IL-6i (tocilizumab, TCZ), and TNFi groups showed a decreased proportion of CX3CR1^+^CD4^+^ T cells, whereas the group treated with a T-cell activation modulator (abatacept, ABT) showed an increased proportion of CX3CR1^+^CD4^+^ T cells (Fig. 5c). Of the two cases treated with abatacept, one showed improvement in arthritis disease activity, while the other still had persistent arthritis activity. A similar trend was observed in the proportion of CX3CR1^+^CD8^+^ T cells (Fig. 5c).

### *PD-1*^+^*CD38*^+^*CX3CR1*^+^*CD4*^+^*T cell is a treatment-resistant T cell subset*

**Fig. 6:**
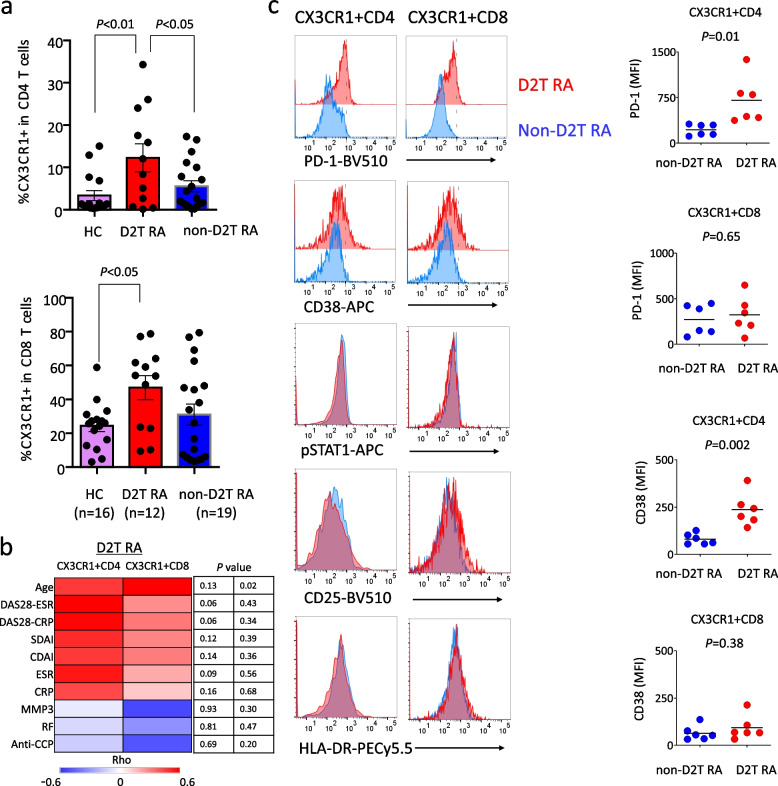
CX3CR1^+^ T cells remain elevated and activated in treatment-resistent RA. **a** Comparative analysis of peripheral blood CX3CR1^+^ CD4^+^ T cells and CX3CR1^+^ CD8^+^ T cells in D2T RA, non-D2T RA, and HC. One-way ANOVA followed by Tukey's multiple comparison test. **b** Heatmap showing correlations between clinical indicators and CX3CR1^+^ CD4^+^ T cells, CX3CR1^+^ CD8^+^ T cells in D2T RA patients. Spearman's correlation analysis. **c** Comparative analysis of activation markers (PD-1, CD38, CD25, HLA-DR) and phosphorylated STAT1 in CX3CR1^+^ T cells between D2T RA (n = 6) and non-D2T RA (n = 6) patients. t test

Finally, we investigated the role of CX3CR1^+^ T cells in D2T RA, a recently recognized treatment-resistant phenotype [16]. We obtained fresh blood samples from 12 patients with D2T RA and 19 patients with non-D2T RA and compared them with samples from 16 healthy controls. As shown in Supplementary Table 2, approximately 50% of the D2T RA patients were LORA, while the remaining 50% were non-LORA. Both CX3CR1^+^CD4^+^ T and CX3CR1^+^CD8^+^ T cells were elevated in the peripheral blood of patients with D2T RA, with CX3CR1^+^CD4^+^ T cells particularly elevated compared with those in patients with non-D2T RA (Fig. 6a). CX3CR1^+^CD4^+^ T cells were found to be positively correlated with arthritis activity and age in patients with D2T RA (Fig. 6b). Notably, CX3CR1^+^CD8^+^ T cells were not associated with arthritis activity in untreated RA overall or in untreated LORA (Fig. 5a) but were correlated with arthritis activity in the D2T RA population (Fig. 6b). Furthermore, we extensively investigated the activation state of CX3CR1^+^ T cells using blood samples from six additional patients with D2T RA. The increased CX3CR1^+^ T cells in the D2T RA group did not show significant activation of the JAK-STAT pathway, nor did they exhibit changes in the expression levels of CD25 or HLA-DR compared with those in non-D2T RA group (Fig. 6c). However, the expression levels of PD-1 and CD38 were significantly elevated in CX3CR1^+^CD4^+^ T cells in the D2T RA group compared with non-D2T RA group (Fig. 6c), suggesting these molecules as treatment-resistant T cell subset markers. These cells accounted for an average of 45% (range: 16.6%–62.7%) of CX3CR1⁺CD4⁺ T cells in D2T RA.

## Discussion

The involvement of CX3CR1^+^ T cells in the pathogenesis of RA was reported in a past study, but how they contribute to the disease pathology was unclear [28]. In the present study, we found that CX3CR1^+^CD4^+^ T cells contribute to synovitis in LORA. CX3CR1^+^CD4^+^ T cells were identified as a cytotoxic ThA subset. These cells were suggested to differentiate in enlarged lymph nodes, migrate into peripheral blood, and subsequently infiltrate synovial tissue. The number of these cells decreased after treatment with methotrexate, TNFi, and IL-6i, but not with T-cell activation modulators. Moreover, in patients with D2T RA, the number of CX3CR1^+^ T cells persistently increased and PD-1^+^CD38^+^CX3CR1^+^ T cells were identified as a treatment-resistant T cell subset. These findings advance the understanding of the pathophysiology of age-associated autoimmune arthritis and treatment-resistant clinical phenotype.

In age-associated T cells, the expression levels of the co-stimulatory molecules CD27, CD28, and CD96 are decreased, while the expression levels of NK cell-related molecules such as CD57 and the adapter molecule CD95 (FAS) are increased [20–23]. We confirmed that CX3CR1^+^T cells have low expression levels of CD27, CD28, and CD96 and high expression levels of CD57, leading us to consider them age-associated T cells. Consistent with these age-associated molecular changes, a positive correlation between age and increased number of CX3CR1^+^ T cells in patients with RA were observed. Intriguingly, we discovered that the expression level of CD95 on CX3CR1^+^ age-associated T cells is not high but “mid”. Past studies have reported that age-associated T cells exhibit resistance to Fas-mediated apoptosis [23]; this may be related to our observation that the expression level of CD95 is not high but “mid.”

As mentioned above, CX3CR1^+^CD4^+^ T cells and CX3CR1^+^CD8^+^ T cells share common molecular features with age-associated T cells. However, CX3CR1^+^CD4^+^ T cells and CX3CR1^+^CD8^+^ T cells were not at the same differentiation stage. Specifically, we identified a novel difference; CX3CR1^+^CD4^+^ T cells were identified EM, whereas CX3CR1^+^CD8^+^ T cells were identified TEMRA. T cells undergo terminal differentiation through various stages, from naïve T cells to central memory, EM, and TEMRA cells, where the proliferative capacity decreases and cytotoxic function increases [21]. Considering that TEMRA is the more advanced stage of differentiation, the susceptibility of human CD4^+^ and CD8^+^ T cells to undergo age-associated changes may vary, with CD8^+^ T cells developing an age-associated phenotype more rapidly than the CD4^+^ T cell population [29, 30]. Further research is necessary to understand the reason for different differentiation stages of CX3CR1^+^CD4^+^ T cells and CX3CR1^+^CD8^+^ T cells within the same individual, along with the physiological significance of these findings.

In humans, specific cell surface markers for age-associated T cells have not yet been identified. In this study, we identified distinctive expression patterns of cell-surface chemokine receptors in CX3CR1^+^ age-associated T cells. Specifically, CX3CR1^+^ age-associated T cells exhibited reduced expression of CCR7, which is necessary for homing to lymphoid tissues and for their retention there [31], while they expressed CX3CR1, which is involved in the migration to peripheral inflammatory tissues. Notably, CX3CR1^+^ age-associated T cells did not express markers of other T cell subsets, such as Th1, Th2, Th17, Tfh, and Treg, and were found to be CXCR3^mid^, consistent with recently reported characteristics of age-associated T cells [25]. These findings suggest that CX3CR1 may serve as a human-specific marker of age-associated T cells.

Thymus, which is a site of T cell differentiation, undergoes involution with age [32]. However, in this study, we observed an increase in the number of CX3CR1^+^ age-associated T cells with age, suggesting that these cells may have differentiated outside of the thymus. In our study, we discovered that LORA patients exhibited primarily mediastinal or axillary lymph node enlargement more frequently than non-LORA patients and confirmed the presence of CX3CR1^+^ age-associated T cells in enlarged lymph nodes. These results suggest a novel perspective that the lymph nodes, rather than the thymus, may serve as the site of differentiation and development of CX3CR1^+^ age-associated T cells.

In patients with RA, an increase in CX3CR1^+^ age-associated T cells was not associated with autoantibody production or sex but was associated with LORA. Specifically, among CX3CR1^+^ age-associated T cells, an increase in CD4^+^ T cells reflects the activity of arthritis and the degree of inflammatory markers. On the other hand, CX3CR1^+^CD8^+^ T cells were associated with aging in RA patients but were not increased compared to healthy individuals and showed no correlation with disease activity. Furthermore, post-treatment analysis of RA showed a decrease in CX3CR1^+^CD4^+^ T cells along with an improvement in arthritis, whereas no significant changes were observed in CX3CR1^+^CD8^+^ T cells. Therefore, CX3CR1^+^CD4^+^ T cells but not CX3CR1^+^CD8^+^ T cells may be associated with disease severity, particularly in patients with LORA. Consistent with our findings, a recent study reported the clonal expansion of cytotoxic CD4^+^ T cells at disease flare compared with that in the remission state, suggesting that cytotoxic CD4^+^ T cells are pathogenic [10]. Cytotoxic CD4^+^ T cells have been reported to undergo chronic antigen-driven expansion [33]; therefore, identifying the causative antigens in LORA can contribute to a deeper understanding of its pathogenesis.

In treatment-specific analyses, a decrease in CX3CR1^+^CD4^+^ T cells was observed in the majority of patients treated with TNFi or IL-6i, but not in those treated with the T-cell activation modulator, abatacept. Abatacept targets CD28, which may have a limited effect on CX3CR1^+^CD4^+^ T cells that do not express CD28. In contrast, a decrease in CD28^−^CD4^+^ T cells following TNFi treatment has been previously reported [34–36], which is consistent with our findings. There are still limited studies examining differences in the effectiveness of each biological disease-modifying antirheumatic drug for LORA, with only two observational studies available. Both observational studies found that the retention rates of other biological disease-modifying antirheumatic drugs were higher compared to abatacept [37, 38]. Therefore, TNFi or IL-6i may be more effective than abatacept in treating LORA. Future clinical studies are needed to determine which biologics are the most effective for the treatment of patients with LORA.

In D2T RA, several drugs with different modes of action are insufficient to control arthritis, which presents a clinically significant challenge [16, 39]. Therefore, improving our understanding of its pathophysiology is essential for the development of new therapeutic strategies. Prior to the current study, the pathogenesis of D2T RA was completely unclear owing to the lack of reported findings. We found that arthritis activity in the D2T RA group correlated with the proportion of CX3CR1^+^CD4^+^ T cells. Furthermore, PD1^+^CD38^+^CX3CR1^+^CD4^+^ T cells were identified as a treatment-resistant T cell subset. PD1^+^CD38^+^CX3CR1^+^CD4^+^ T cells are likely a subset of CX3CR1^+^CD4^+^ T cells that have undergone additional activation and possibly further differentiation in response to chronic inflammatory conditions, as both PD-1 and CD38 are known markers of chronic activation [40, 41]. Notably, half of the D2T RA cases in this study were non-LORA, suggesting that factors independent of aging contribute to the differentiation of PD1^+^CD38^+^CX3CR1^+^CD4^+^ T cells. Previous studies suggest that type I interferon may serve as a differentiation-inducing factor for these cells [42, 43]. Therefore, these cells may evade the effects of TNF-α and IL-6 inhibition because their function is not solely driven by TNF-α or IL-6 signaling, but also by other pathways, including the type I interferon signaling. PD-1⁺CD38⁺CX3CR1⁺CD4⁺ T cells resemble the recently reported Tph2 cells and are known to produce not only cytotoxic molecules but also cytokines such as interferon-γ and chemokines such as CCL3 and CCL4 [44]. Future large-scale prospective studies are warranted to determine whether the presence of increased PD1^+^CD38^+^CX3CR1^+^CD4^+^ T cells before treatment initiation can serve as a predictive marker for future treatment resistance.

Specific optimal treatment strategies for LORA and D2T RA are not currently established. E6011, a novel cell trafficking inhibitor targeting the human fractalkine-CX3CR1 interaction, demonstrated significant therapeutic effects at week 24, although it did not achieve the primary endpoint at week 12 in RA [45, 46]. Considering the involvement of CX3CR1 in the pathogenesis of LORA and D2T RA, future clinical studies are needed to determine the therapeutic efficacy of E6011, specifically in LORA and D2T RA.

## Conclusions

Patients with LORA present distinct immunological characteristics, including increased numbers of CX3CR1^+^ cytotoxic CD4^+^ T cells displaying age-associated features. These cells may originate in the enlarged lymph nodes, and infiltrate the synovial tissues. Furthermore, we identified PD1^+^CD38^+^CX3CR1^+^CD4^+^ T cells as a treatment-resistant T cell subset. Our findings provide insights into age-associated autoimmune arthritis and potential therapeutic targets.

## Supplementary Information

Supplementary Material 1.

## Data Availability

All data generated or analysed during this study are included in this published article.

## Abbreviations

RA: Rheumatoid Arthritis
LORA: Late-Onset Rheumatoid Arthritis
D2T RA: Difficult-to-Treat Rheumatoid Arthritis
TNFi: Tumor Necrosis Factor Inhibitors
IL-6i: Interleukin-6 Inhibitors
ThA: Age-Associated T Helper Cells
EM: Effector Memory
TEMRA: Terminally Differentiated Effector Memory T Cells Re-expressing CD45RA
GZMB: Granzyme B
STAT1: Signal Transducer and Activator of Transcription 1
CDAI: Clinical Disease Activity Index
DAS28: Disease Activity Score 28-joint count
SDAI: Simplified Disease Activity Index
ABT: Abatacept
TCZ: Tocilizumab

## References

[1] McInnes IB, Schett G. The pathogenesis of rheumatoid arthritis. N Engl J Med. 2011;365:2205–19. 22150039 10.1056/NEJMra1004965

[2] Firestein GS, McInnes IB. Immunopathogenesis of rheumatoid arthritis. Immunity. 2017;46:183–96. 28228278 10.1016/j.immuni.2017.02.006 PMC5385708

[3] Gravallese EM, Firestein GS. Rheumatoid Arthritis - Common Origins. Divergent Mechanisms N Engl J Med. 2023;388:529–42. 36780677 10.1056/NEJMra2103726

[4] Malmström V, Catrina AI, Klareskog L. The immunopathogenesis of seropositive rheumatoid arthritis: from triggering to targeting. Nat Rev Immunol. 2017;17:60–75. 27916980 10.1038/nri.2016.124

[5] Smolen JS, Aletaha D, Barton A, Burmester GR, Emery P, Firestein GS, et al. Rheumatoid arthritis Nat Rev Dis Primers. 2018;4:18001. 29417936 10.1038/nrdp.2018.1

[6] Ishigaki K, Lagattuta KA, Luo Y, James EA, Buckner JH, Raychaudhuri S. HLA autoimmune risk alleles restrict the hypervariable region of T cell receptors. Nat Genet. 2022;54:393–402. 35332318 10.1038/s41588-022-01032-z PMC9010379

[7] Ha E, Bae S, Kim K. Large-scale meta-analysis across East Asian and European populations updated genetic architecture and variant-driven biology of rheumatoid arthritis, identifying 11 novel susceptibility loci. Ann Rheum Dis. 2021;80:558–65. 33310728 10.1136/annrheumdis-2020-219065 PMC8053349

[8] Rao DA, Gurish MF, Marshall JL, Slowikowski K, Fonseka CY, Liu Y, et al. Pathologically expanded peripheral T helper cell subset drives B cells in rheumatoid arthritis. Nature. 2017;542:110–4. 28150777 10.1038/nature20810 PMC5349321

[9] Akiyama M, Alshehri W, Yoshimoto K, Kaneko Y. T follicular helper cells and T peripheral helper cells in rheumatic and musculoskeletal diseases. Ann Rheum Dis. 2023;82:1371–81. 37414520 10.1136/ard-2023-224225

[10] Baker KF, McDonald D, Hulme G, Hussain R, Coxhead J, Swan D, et al. Single-cell insights into immune dysregulation in rheumatoid arthritis flare versus drug-free remission. Nat Commun. 2024;15:1063. 38316770 10.1038/s41467-024-45213-2 PMC10844292

[11] Argyriou A, Wadsworth MH 2nd, Lendvai A, Christensen SM, Hensvold AH, Gerstner C, et al. Single cell sequencing identifies clonally expanded synovial CD4(+) T(PH) cells expressing GPR56 in rheumatoid arthritis. Nat Commun. 2022;13:4046. 35831277 10.1038/s41467-022-31519-6 PMC9279430

[12] Boots AM, Maier AB, Stinissen P, Masson P, Lories RJ, De Keyser F. The influence of ageing on the development and management of rheumatoid arthritis. Nat Rev Rheumatol. 2013;9:604–13. 23774902 10.1038/nrrheum.2013.92

[13] Arnett FC, Edworthy SM, Bloch DA, McShane DJ, Fries JF, Cooper NS, et al. The American Rheumatism Association 1987 revised criteria for the classification of rheumatoid arthritis. Arthritis Rheum. 1988;31:315–24. 3358796 10.1002/art.1780310302

[14] Aletaha D, Neogi T, Silman AJ, Funovits J, Felson DT, Bingham CO 3rd, et al. 2010 rheumatoid arthritis classification criteria: an American College of Rheumatology/European League Against Rheumatism collaborative initiative. Ann Rheum Dis. 2010;69:1580–8. 20699241 10.1136/ard.2010.138461

[15] Yukioka M, Wakitani S, Murata N, Toda Y, Ogawa R, Kaneshige T, et al. Elderly-onset rheumatoid arthritis and its association with HLA-DRB1 alleles in Japanese. Br J Rheumatol. 1998;37:98–101. 9487258 10.1093/rheumatology/37.1.98

[16] Nagy G, Roodenrijs NMT, Welsing PM, Kedves M, Hamar A, van der Goes MC, et al. EULAR definition of difficult-to-treat rheumatoid arthritis. Ann Rheum Dis. 2021;80:31–5. 33004335 10.1136/annrheumdis-2020-217344 PMC7788062

[17] el Amir AD, Davis KL, Tadmor MD, Simonds EF, Levine JH, Bendall SC, et al. viSNE enables visualization of high dimensional single-cell data and reveals phenotypic heterogeneity of leukemia. Nat Biotechnol. 2013;31:545–52. 23685480 10.1038/nbt.2594 PMC4076922

[18] Akiyama M, Yoshimoto K, Kaneko Y. Significant association of CX3CR1+CD8 T cells with aging and distinct clinical features in Sjögren’s syndrome and IgG4-related disease. Clin Exp Rheumatol. 2023;41:2409–17. 37812481 10.55563/clinexprheumatol/kfsd65

[19] Volkov M, van Schie KA, van der Woude D. Autoantibodies and B Cells: The ABC of rheumatoid arthritis pathophysiology. Immunol Rev. 2020;294:148–63. 31845355 10.1111/imr.12829 PMC7065213

[20] Kared H, Martelli S, Ng TP, Pender SL, Larbi A. CD57 in human natural killer cells and T-lymphocytes. Cancer Immunol Immunother. 2016;65:441–52. 26850637 10.1007/s00262-016-1803-z PMC11029668

[21] Laphanuwat P, Gomes DCO, Akbar AN. Senescent T cells: Beneficial and detrimental roles. Immunol Rev. 2023;316:160–75. 37098109 10.1111/imr.13206 PMC10952287

[22] Bunet R, Nayrac M, Ramani H, Sylla M, Durand M, Chartrand-Lefebvre C, et al. Loss of CD96 Expression as a Marker of HIV-Specific CD8+ T-Cell Differentiation and Dysfunction. Front Immunol. 2021;12: 673061. 34122431 10.3389/fimmu.2021.673061 PMC8190400

[23] Ettinger R, Panka DJ, Wang JK, Stanger BZ, Ju ST, Marshak-Rothstein A. Fas ligand-mediated cytotoxicity is directly responsible for apoptosis of normal CD4+ T cells responding to a bacterial superantigen. J Immunol. 1995;154:4302–8. 7536768

[24] Maecker HT, McCoy JP, Nussenblatt R. Standardizing immunophenotyping for the Human Immunology Project. Nat Rev Immunol. 2012;12:191–200. 22343568 10.1038/nri3158 PMC3409649

[25] Goto M, Takahashi H, Yoshida R, Itamiya T, Nakano M, Nagafuchi Y, et al. Age-associated CD4+ T cells with B cell-promoting functions are regulated by ZEB2 in autoimmunity. Sci Immunol. 2024;9:eadk1643. 10.1126/sciimmunol.adk1643 38330141

[26] Inoue E, Yamanaka H, Hara M, Tomatsu T, Kamatani N. Comparison of Disease Activity Score (DAS)28- erythrocyte sedimentation rate and DAS28- C-reactive protein threshold values. Ann Rheum Dis. 2007;66:407–9. 16926186 10.1136/ard.2006.054205 PMC1856019

[27] Kaneko Y, Kondo H, Takeuchi T. American College of Rheumatology/European League Against Rheumatism remission criteria for rheumatoid arthritis maintain reliable performance when evaluated in 44 joints. J Rheumatol. 2013;40:1254–8. 23772077 10.3899/jrheum.130166

[28] Nanki T, Imai T, Nagasaka K, Urasaki Y, Nonomura Y, Taniguchi K, et al. Migration of CX3CR1-positive T cells producing type 1 cytokines and cytotoxic molecules into the synovium of patients with rheumatoid arthritis. Arthritis Rheum. 2002;46:2878–83. 12428227 10.1002/art.10622

[29] Callender LA, Carroll EC, Bober EA, Akbar AN, Solito E, Henson SM. Mitochondrial mass governs the extent of human T cell senescence. Aging Cell. 2020;19: e13067. 31788930 10.1111/acel.13067 PMC6996952

[30] Czesnikiewicz-Guzik M, Lee WW, Cui D, Hiruma Y, Lamar DL, Yang ZZ, et al. T cell subset-specific susceptibility to aging. Clin Immunol. 2008;127:107–18. 18222733 10.1016/j.clim.2007.12.002 PMC2435295

[31] Cuesta-Mateos C, Brown JR, Terrón F, Muñoz-Calleja C. Of Lymph Nodes and CLL Cells: Deciphering the Role of CCR7 in the Pathogenesis of CLL and Understanding Its Potential as Therapeutic Target. Front Immunol. 2021;12: 662866. 33841445 10.3389/fimmu.2021.662866 PMC8024566

[32] Prelog M. Aging of the immune system: a risk factor for autoimmunity? Autoimmun Rev. 2006;5:136–9. 16431345 10.1016/j.autrev.2005.09.008

[33] Cenerenti M, Saillard M, Romero P, Jandus C. The Era of Cytotoxic CD4 T Cells. Front Immunol. 2022;13: 867189. 35572552 10.3389/fimmu.2022.867189 PMC9094409

[34] Gerli R, Schillaci G, Giordano A, Bocci EB, Bistoni O, Vaudo G, et al. CD4+CD28- T lymphocytes contribute to early atherosclerotic damage in rheumatoid arthritis patients. Circulation. 2004;109:2744–8. 15159291 10.1161/01.CIR.0000131450.66017.B3

[35] Bryl E, Vallejo AN, Matteson EL, Witkowski JM, Weyand CM, Goronzy JJ. Modulation of CD28 expression with anti-tumor necrosis factor alpha therapy in rheumatoid arthritis. Arthritis Rheum. 2005;52:2996–3003. 16200579 10.1002/art.21353

[36] Pawlik A, Ostanek L, Brzosko I, Brzosko M, Masiuk M, Machalinski B, et al. Therapy with infliximab decreases the CD4+CD28- T cell compartment in peripheral blood in patients with rheumatoid arthritis. Rheumatol Int. 2004;24:351–4. 14504910 10.1007/s00296-003-0374-4

[37] Jinno S, Onishi A, Hattori S, Dubreuil M, Ueda Y, Nishimura K, et al. Comparison of retention of biologics in Japanese patients with elderly-onset rheumatoid arthritis-the ANSWER cohort study. Rheumatology (Oxford). 2024 Feb 6:keae081. 10.1093/rheumatology/keae081. Online ahead of print. 10.1093/rheumatology/keae081 38317442

[38] Jeries H, Daood R, Hijazi B, Golan-Cohen A, Green I, Merzon E, et al. Drug Survival on First Biologic Therapy Among Late-Onset Rheumatoid Arthritis Patients Compared to Early-Onset Patients: A Population-Based Cohort Study. Musculoskeletal Care. 2024;22: e1928. 39152548 10.1002/msc.1928

[39] Nagy G, Roodenrijs NMT, Welsing PMJ, Kedves M, Hamar A, van der Goes MC, et al. EULAR points to consider for the management of difficult-to-treat rheumatoid arthritis. Ann Rheum Dis. 2022;81:20–33. 34407926 10.1136/annrheumdis-2021-220973 PMC8761998

[40] Zou W, Wolchok JD, Chen L. PD-L1 (B7-H1) and PD-1 pathway blockade for cancer therapy: Mechanisms, response biomarkers, and combinations. Sci Transl Med. 2016;8:328rv4. 10.1126/scitranslmed.aad7118 PMC4859220 26936508

[41] Quarona V, Zaccarello G, Chillemi A, Brunetti E, Singh VK, Ferrero E, et al. CD38 and CD157: a long journey from activation markers to multifunctional molecules. Cytometry B Clin Cytom. 2013;84:207–17. 23576305 10.1002/cyto.b.21092

[42] Tanemura S, Tsujimoto H, Seki N, Kojima S, Miyoshi F, Sugahara K, et al. Role of interferons (IFNs) in the differentiation of T peripheral helper (Tph) cells. Int Immunol. 2022;34:519–32. 35723683 10.1093/intimm/dxac026

[43] Wang R, Singaraju A, Marks KE, Shakib L, Dunlap G, Adejoorin I, et al. Clonally expanded CD38hi cytotoxic CD8 T cells define the T cell infiltrate in checkpoint inhibitor-associated arthritis. Sci Immunol. 2023;8:eadd1591. 10.1126/sciimmunol.add1591 PMC10557056 37506196

[44] Seki N, Tsujimoto H, Tanemura S, Kojima S, Miyoshi F, Kikuchi J, et al. Cytotoxic Tph subset with low B-cell helper functions and its involvement in systemic lupus erythematosus. Commun Biol. 2024;7:277. 38448723 10.1038/s42003-024-05989-x PMC10918188

[45] Tanaka Y, Takeuchi T, Yamanaka H, Nanki T, Umehara H, Yasuda N, et al. Efficacy and Safety of E6011, an Anti-Fractalkine Monoclonal Antibody, in Patients With Active Rheumatoid Arthritis With Inadequate Response to Methotrexate: Results of a Randomized, Double-Blind. Placebo-Controlled Phase II Study Arthritis Rheumatol. 2021;73:587–95. 33038062 10.1002/art.41555 PMC8048525

[46] Tanaka Y, Takeuchi T, Yamanaka H, Nanki T, Umehara H, Yasuda N, et al. Long-term safety and efficacy of E6011, an anti-fractalkine monoclonal antibody, in patients with rheumatoid arthritis inadequately responding to methotrexate. Mod Rheumatol. 2023;34:37–44. 36680426 10.1093/mr/road004

