# Supplementary material for: CX3CR1+ age-associated CD4+ T cells contribute to synovial inflammation in late-onset rheumatoid arthritis
Source: Inflamm Regen. 2025 Feb 6;45:4. doi: 10.1186/s41232-025-00367-4 (PMC11800492; doi:10.1186/s41232-025-00367-4)
Supplement: Supplementary file 1 — Supplementary Material 1. [file 41232_2025_367_MOESM1_ESM.pptx]

## Slide 1
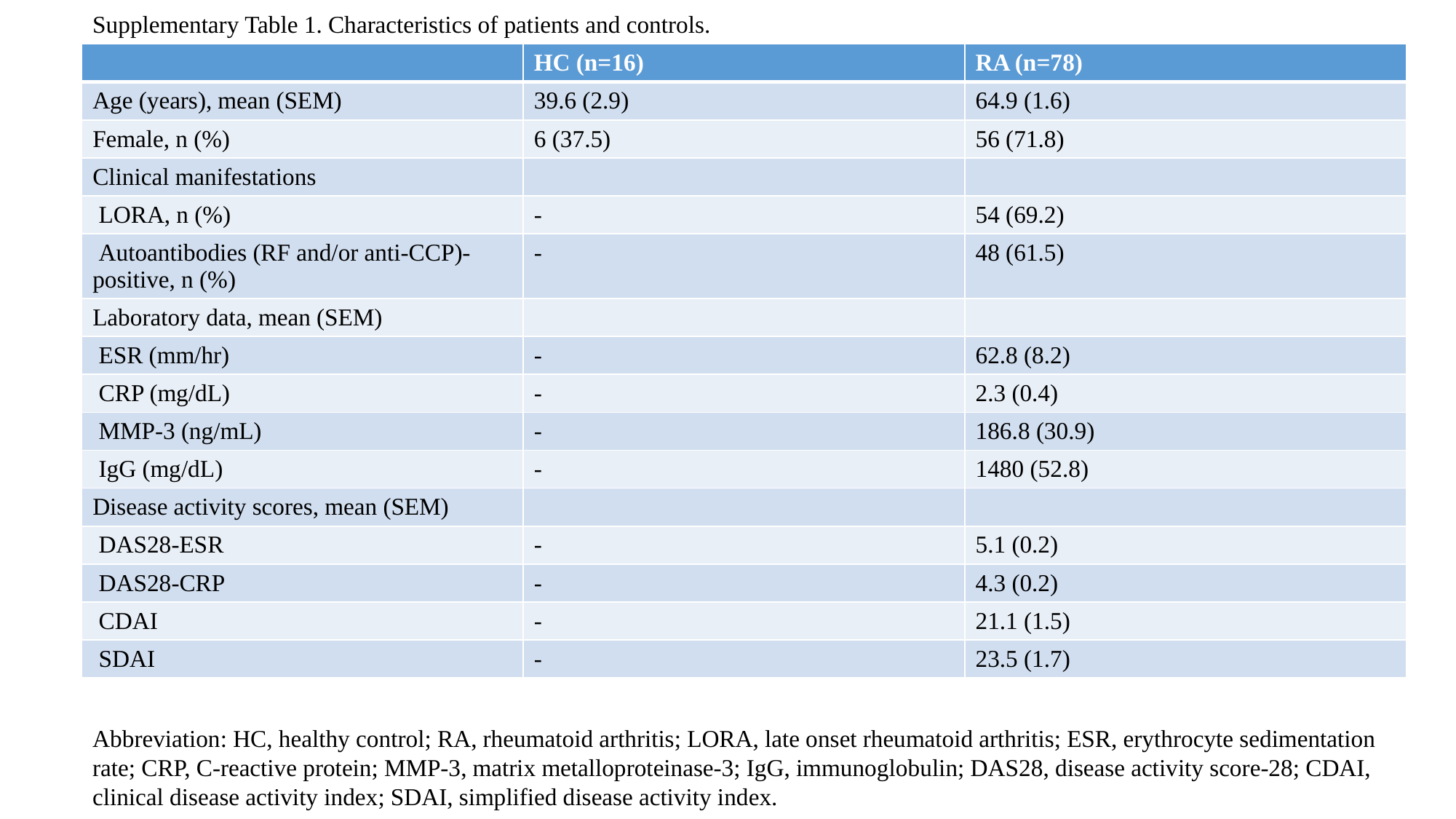

Supplementary Table 1. Characteristics of patients and controls.
| | HC (n=16) | RA (n=78) |
| --- | --- | --- |
| Age (years), mean (SEM) | 39.6 (2.9) | 64.9 (1.6) |
| Female, n (%) | 6 (37.5) | 56 (71.8) |
| Clinical manifestations | | |
| LORA, n (%) | - | 54 (69.2) |
| Autoantibodies (RF and/or anti-CCP)-positive, n (%) | - | 48 (61.5) |
| Laboratory data, mean (SEM) | | |
| ESR (mm/hr) | - | 62.8 (8.2) |
| CRP (mg/dL) | - | 2.3 (0.4) |
| MMP-3 (ng/mL) | - | 186.8 (30.9) |
| IgG (mg/dL) | - | 1480 (52.8) |
| Disease activity scores, mean (SEM) | | |
| DAS28-ESR | - | 5.1 (0.2) |
| DAS28-CRP | - | 4.3 (0.2) |
| CDAI | - | 21.1 (1.5) |
| SDAI | - | 23.5 (1.7) |
Abbreviation: HC, healthy control; RA, rheumatoid arthritis; LORA, late onset rheumatoid arthritis; ESR, erythrocyte sedimentation rate; CRP, C-reactive protein; MMP-3, matrix metalloproteinase-3; IgG, immunoglobulin; DAS28, disease activity score‐28; CDAI, clinical disease activity index; SDAI, simplified disease activity index.

## Slide 2
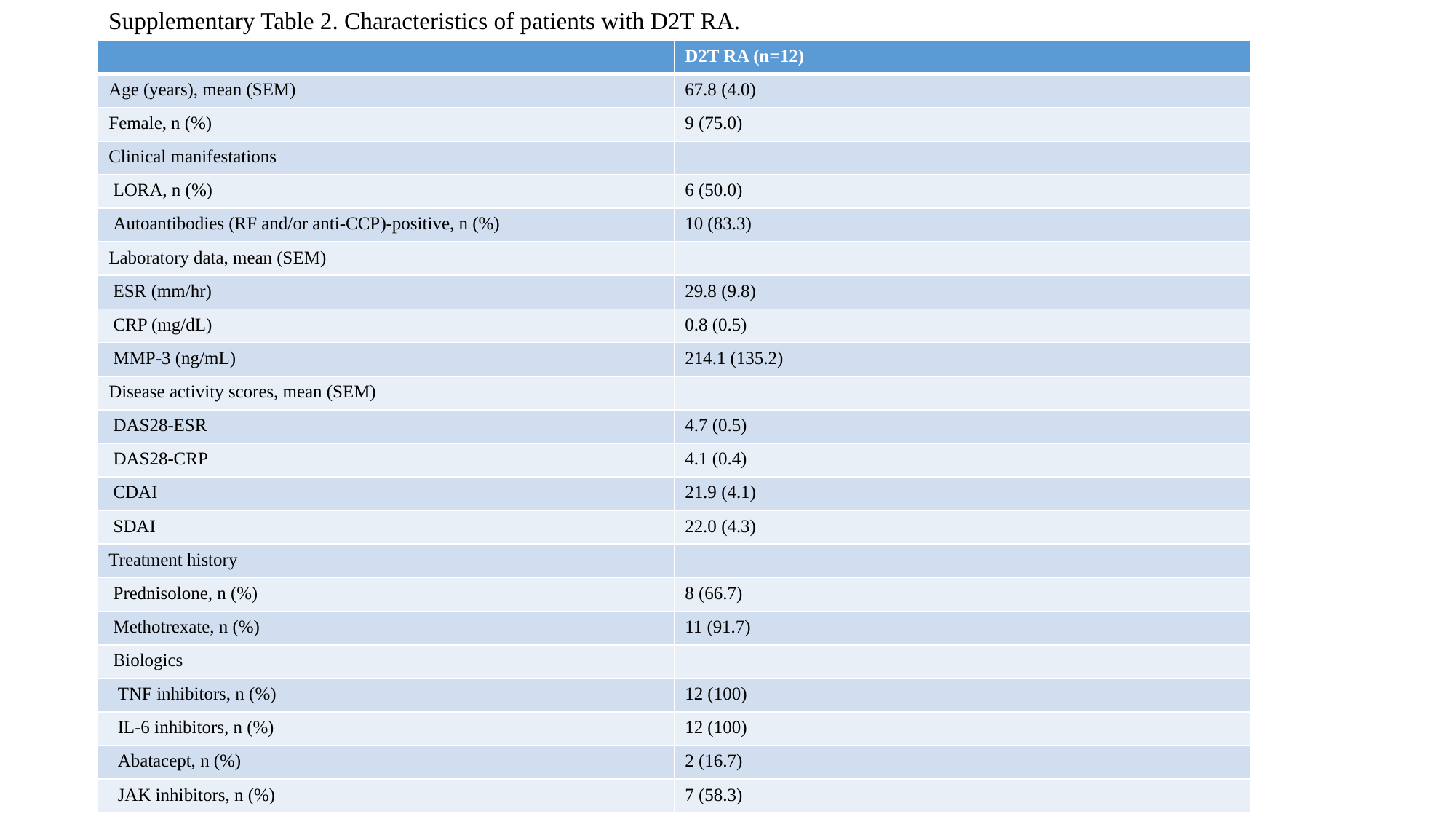

Supplementary Table 2. Characteristics of patients with D2T RA.
| | D2T RA (n=12) |
| --- | --- |
| Age (years), mean (SEM) | 67.8 (4.0) |
| Female, n (%) | 9 (75.0) |
| Clinical manifestations | |
| LORA, n (%) | 6 (50.0) |
| Autoantibodies (RF and/or anti-CCP)-positive, n (%) | 10 (83.3) |
| Laboratory data, mean (SEM) | |
| ESR (mm/hr) | 29.8 (9.8) |
| CRP (mg/dL) | 0.8 (0.5) |
| MMP-3 (ng/mL) | 214.1 (135.2) |
| Disease activity scores, mean (SEM) | |
| DAS28-ESR | 4.7 (0.5) |
| DAS28-CRP | 4.1 (0.4) |
| CDAI | 21.9 (4.1) |
| SDAI | 22.0 (4.3) |
| Treatment history | |
| Prednisolone, n (%) | 8 (66.7) |
| Methotrexate, n (%) | 11 (91.7) |
| Biologics | |
| TNF inhibitors, n (%) | 12 (100) |
| IL-6 inhibitors, n (%) | 12 (100) |
| Abatacept, n (%) | 2 (16.7) |
| JAK inhibitors, n (%) | 7 (58.3) |

## Slide 3
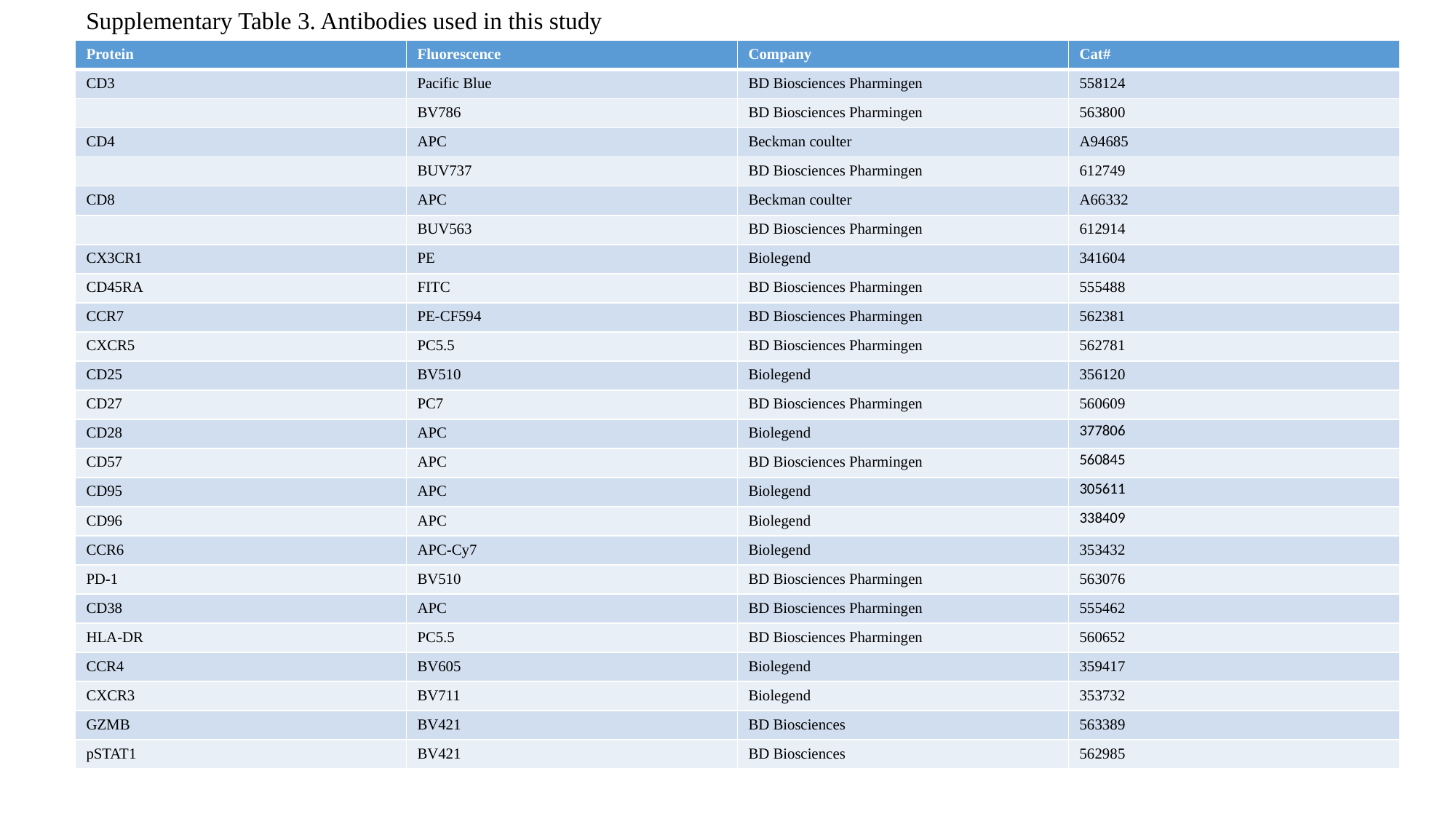

Supplementary Table 3. Antibodies used in this study
| Protein | Fluorescence | Company | Cat# |
| --- | --- | --- | --- |
| CD3 | Pacific Blue | BD Biosciences Pharmingen | 558124 |
| | BV786 | BD Biosciences Pharmingen | 563800 |
| CD4 | APC | Beckman coulter | A94685 |
| | BUV737 | BD Biosciences Pharmingen | 612749 |
| CD8 | APC | Beckman coulter | A66332 |
| | BUV563 | BD Biosciences Pharmingen | 612914 |
| CX3CR1 | PE | Biolegend | 341604 |
| CD45RA | FITC | BD Biosciences Pharmingen | 555488 |
| CCR7 | PE-CF594 | BD Biosciences Pharmingen | 562381 |
| CXCR5 | PC5.5 | BD Biosciences Pharmingen | 562781 |
| CD25 | BV510 | Biolegend | 356120 |
| CD27 | PC7 | BD Biosciences Pharmingen | 560609 |
| CD28 | APC | Biolegend | 377806 |
| CD57 | APC | BD Biosciences Pharmingen | 560845 |
| CD95 | APC | Biolegend | 305611 |
| CD96 | APC | Biolegend | 338409 |
| CCR6 | APC-Cy7 | Biolegend | 353432 |
| PD-1 | BV510 | BD Biosciences Pharmingen | 563076 |
| CD38 | APC | BD Biosciences Pharmingen | 555462 |
| HLA-DR | PC5.5 | BD Biosciences Pharmingen | 560652 |
| CCR4 | BV605 | Biolegend | 359417 |
| CXCR3 | BV711 | Biolegend | 353732 |
| GZMB | BV421 | BD Biosciences | 563389 |
| pSTAT1 | BV421 | BD Biosciences | 562985 |

## Slide 4
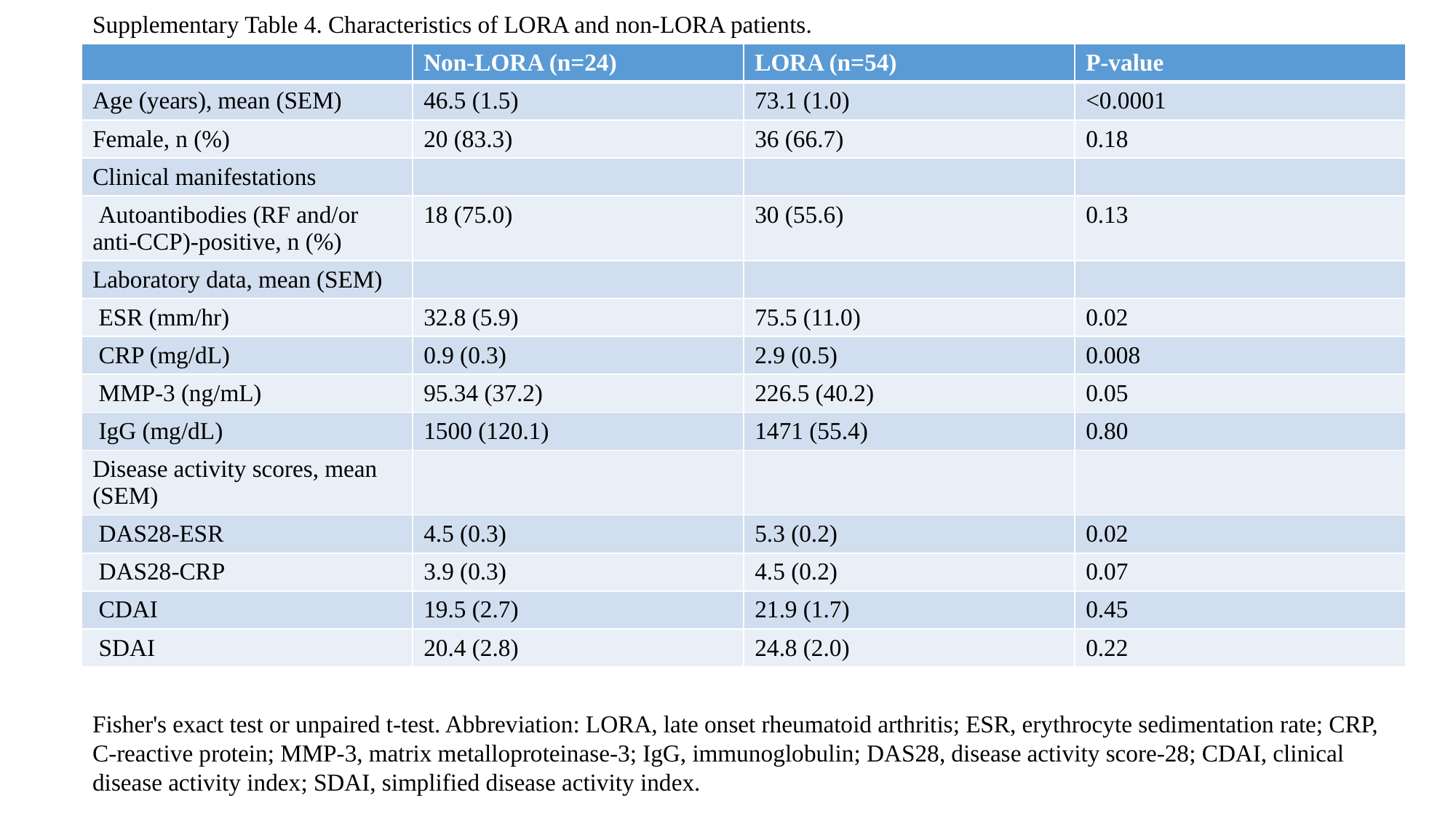

Supplementary Table 4. Characteristics of LORA and non-LORA patients.
| | Non-LORA (n=24) | LORA (n=54) | P-value |
| --- | --- | --- | --- |
| Age (years), mean (SEM) | 46.5 (1.5) | 73.1 (1.0) | <0.0001 |
| Female, n (%) | 20 (83.3) | 36 (66.7) | 0.18 |
| Clinical manifestations | | | |
| Autoantibodies (RF and/or anti-CCP)-positive, n (%) | 18 (75.0) | 30 (55.6) | 0.13 |
| Laboratory data, mean (SEM) | | | |
| ESR (mm/hr) | 32.8 (5.9) | 75.5 (11.0) | 0.02 |
| CRP (mg/dL) | 0.9 (0.3) | 2.9 (0.5) | 0.008 |
| MMP-3 (ng/mL) | 95.34 (37.2) | 226.5 (40.2) | 0.05 |
| IgG (mg/dL) | 1500 (120.1) | 1471 (55.4) | 0.80 |
| Disease activity scores, mean (SEM) | | | |
| DAS28-ESR | 4.5 (0.3) | 5.3 (0.2) | 0.02 |
| DAS28-CRP | 3.9 (0.3) | 4.5 (0.2) | 0.07 |
| CDAI | 19.5 (2.7) | 21.9 (1.7) | 0.45 |
| SDAI | 20.4 (2.8) | 24.8 (2.0) | 0.22 |
Fisher's exact test or unpaired t-test. Abbreviation: LORA, late onset rheumatoid arthritis; ESR, erythrocyte sedimentation rate; CRP, C-reactive protein; MMP-3, matrix metalloproteinase-3; IgG, immunoglobulin; DAS28, disease activity score‐28; CDAI, clinical disease activity index; SDAI, simplified disease activity index.

## Slide 5
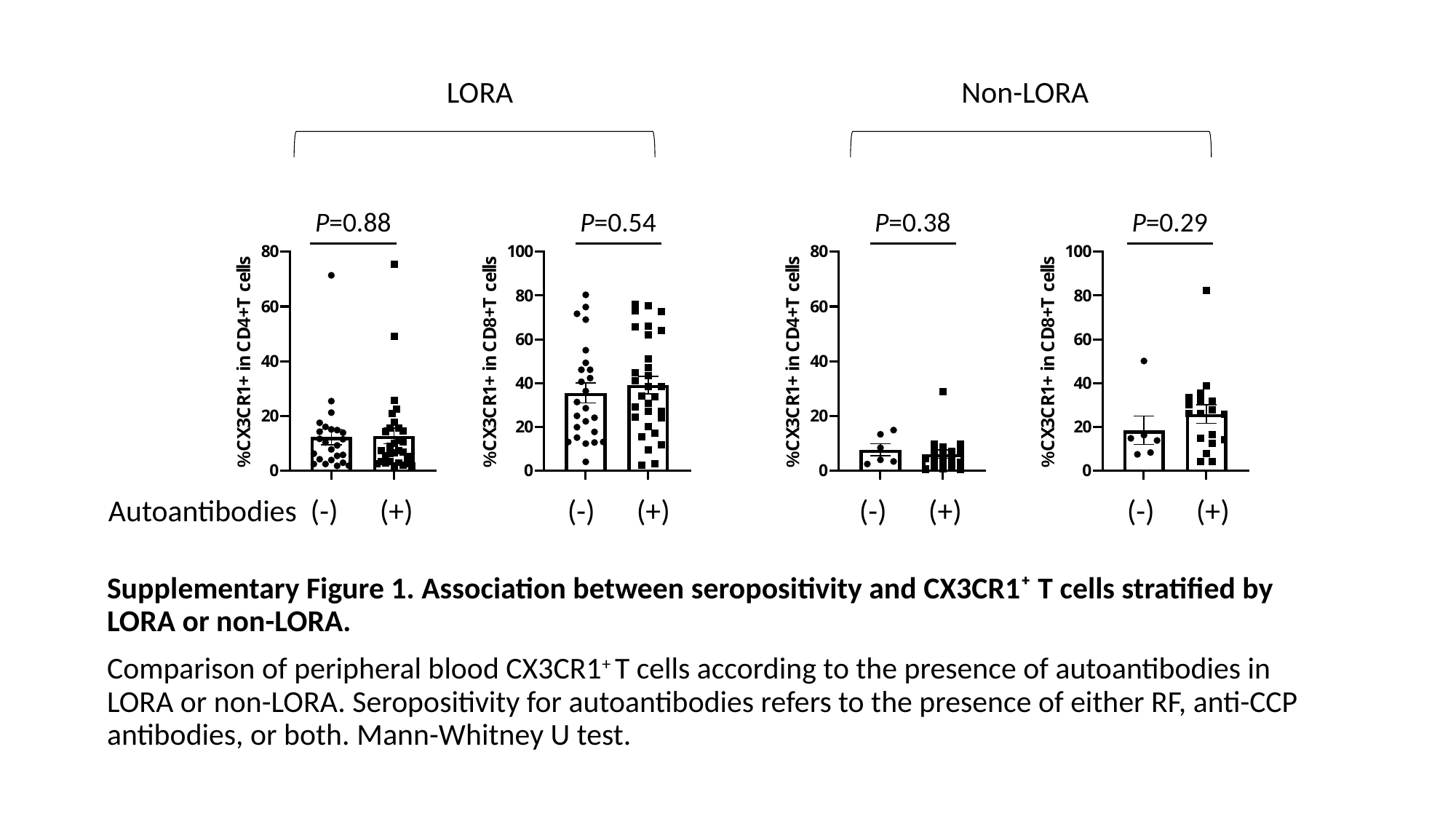

LORA
Non-LORA
P=0.88
P=0.54
P=0.38
P=0.29
 (-) (+)
Autoantibodies (-) (+)
 (-) (+)
 (-) (+)
Supplementary Figure 1. Association between seropositivity and CX3CR1⁺ T cells stratified by LORA or non-LORA.
Comparison of peripheral blood CX3CR1+ T cells according to the presence of autoantibodies in LORA or non-LORA. Seropositivity for autoantibodies refers to the presence of either RF, anti-CCP antibodies, or both. Mann-Whitney U test.

## Slide 6
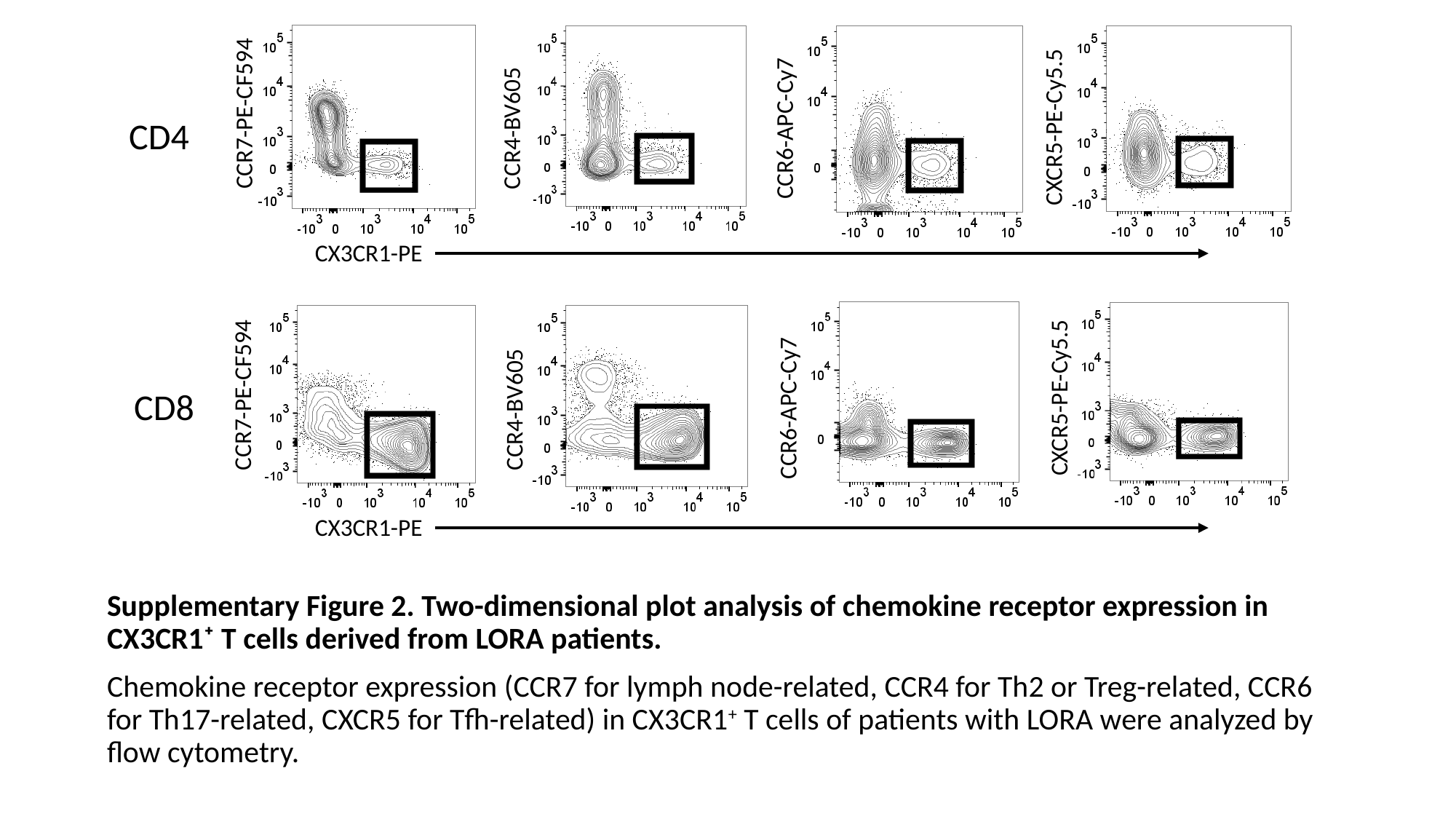

CCR7-PE-CF594
CXCR5-PE-Cy5.5
CD4
CCR4-BV605
CCR6-APC-Cy7
CX3CR1-PE
CCR7-PE-CF594
CXCR5-PE-Cy5.5
CD8
CCR6-APC-Cy7
CCR4-BV605
CX3CR1-PE
Supplementary Figure 2. Two-dimensional plot analysis of chemokine receptor expression in CX3CR1⁺ T cells derived from LORA patients.
Chemokine receptor expression (CCR7 for lymph node-related, CCR4 for Th2 or Treg-related, CCR6 for Th17-related, CXCR5 for Tfh-related) in CX3CR1+ T cells of patients with LORA were analyzed by flow cytometry.

## Slide 7
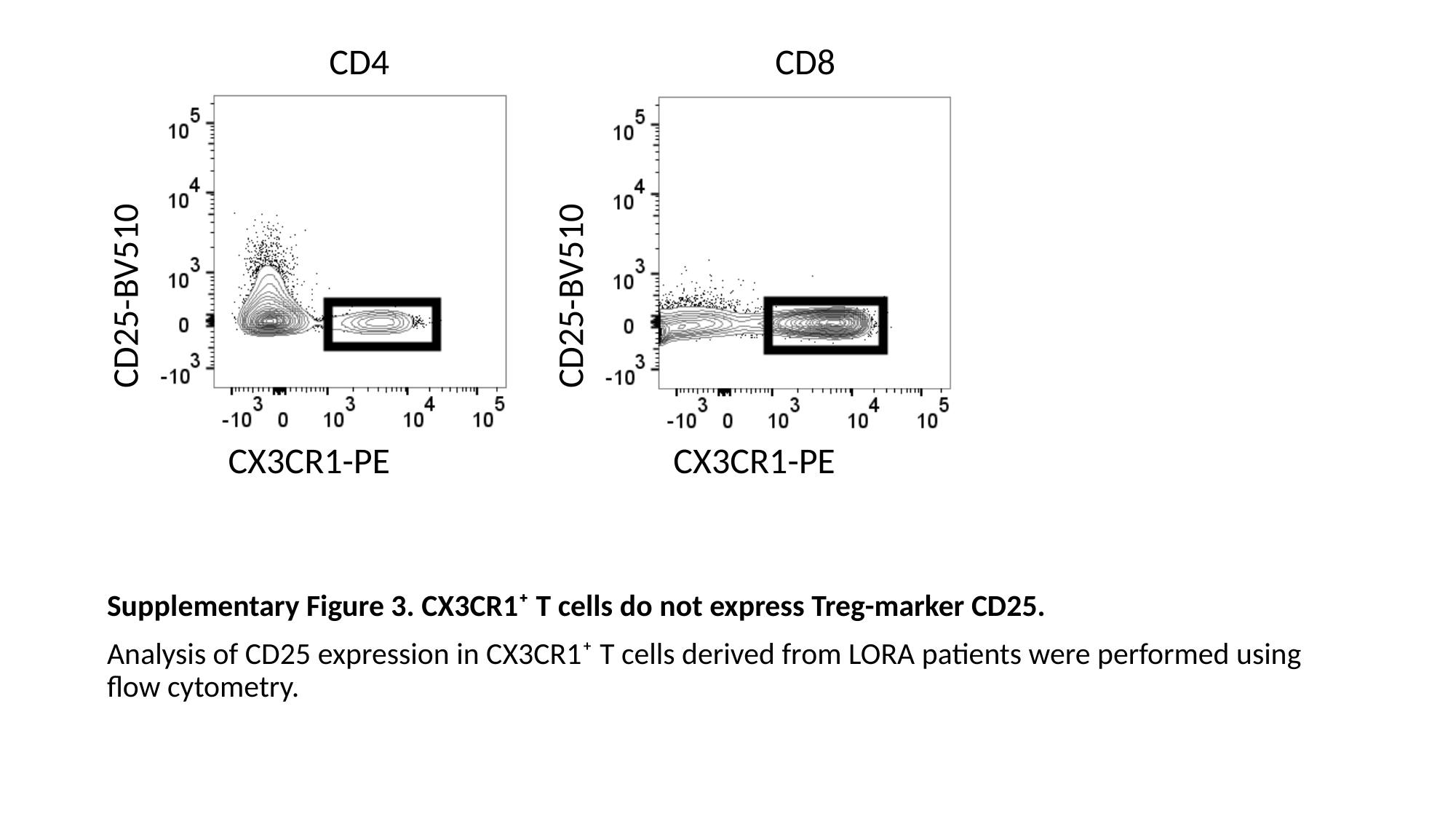

CD4
CD8
CD25-BV510
CD25-BV510
CX3CR1-PE
CX3CR1-PE
Supplementary Figure 3. CX3CR1⁺ T cells do not express Treg-marker CD25.
Analysis of CD25 expression in CX3CR1⁺ T cells derived from LORA patients were performed using flow cytometry.
